# 99164 Resting Functional Connectivity of Networks Associated with Preoccupation in Alcohol Use Disorder Predicts Time to Relapse

**DOI:** 10.1017/cts.2021.508

**Published:** 2021-03-30

**Authors:** Emily M. Koithan, Kai Xuan Nyoi, Timothy Hendrickson, Hannah Verdoorn, Casey Gilmore, Bryon Mueller, Matt Kushner, Kelvin Lim, Jazmin Camchong

**Affiliations:** 1 University of Minnesota Department of Psychiatry and Behavioral Sciences; 2 University of Minnesota Informatics Institute; 3 Minneapolis VA Health Care System

## Abstract

ABSTRACT IMPACT: Our research has the potential to impact human health by identifying a neural network that can be used to predict time to relapse in individuals with alcohol use disorder. OBJECTIVES/GOALS: Preoccupation towards alcohol use (e.g. craving, rumination, and poor executive control) is a maladaptive behavior associated with relapse risk. We investigated whether alterations in resting state networks known to mediate preoccupation could predict time to relapse in alcohol use disorder (AUD). METHODS/STUDY POPULATION: 50 participants with alcohol use disorder (AUD) (Age: M=41.76, SD=10.22, 19 females) were recruited from an addiction treatment program at ˜2 weeks of abstinence. fMRI data were preprocessed with the Human Connectome Project pipeline. Strength of resting state functional connectivity (RSFC) within two networks known to mediate the ‘Preoccupation go’ (PG) and ‘Preoccupation stop’ (PS) stages of addiction were calculated. T-tests were conducted to compare RSFC between subsequent abstainers and relapsers (after 4 months). Linear regressions were conducted to determine whether RSFC (of PG and PS networks) can predict time to relapse. Craving measures were included in the model. RESULTS/ANTICIPATED RESULTS: 19 AUD relapsed during the 4-month follow-up period. There were no RSFC group effects (subsequent abstainers and relapsers) in the PG or PS networks. Number of days to relapse could be predicted by PG RSFC (F(1,17)=14.90, p=0.001, r ^2^=0.47). Time to relapse increased by 13.19 days for each PG RSFC unit increase. Number of days to relapse could be predicted by PS RSFC (F(1,17)=9.39, p=0.002, r ²=0.36). Time to relapse increased by 12.94 days for each PS RSFC unit increase. After adding a self-report craving measure (i.e. Penn Alcohol Craving Scale) in the prediction model, both PG and PS RSFC still significantly predicted time to relapse. Craving metric did not predict time to relapse. DISCUSSION/SIGNIFICANCE OF FINDINGS: RSFC in preoccupation networks during short-term abstinence predicted time to relapse. These preliminary findings highlight promising targets for AUD neuromodulation interventions aimed to reduce relapse. Future larger scale studies that examine the effects of covariates and mediators are needed.

